# Myopia and Regional Variations in Retinal Thickness in Healthy Eyes

**DOI:** 10.18502/jovr.v15i2.6735

**Published:** 2020-04-06

**Authors:** Feryal M. Zereid, Uchechukwu L. Osuagwu

**Affiliations:** ^1^ Department of Optometry & Vision Sciences, College of Applied Medical Sciences, King Saud University, Riyadh, Saudi Arabia; ^2^ School of Medicine, Diabetes Obesity and Metabolism Translational Research Unit (DOMTRU), Macarthur Clinical School, Parkside Crescent, Campbelltown, Australia; ^3^ African Vision Research Institute, University of KwaZulu-Natal, Durban, South Africa

**Keywords:** Fovea, Myopia, Refractive Error, Retinal Thickness, Saudi Arabia, Stratus Optical Coherence Tomography

## Abstract

**Purpose:**

To investigate the effects of refraction on retinal thickness measurements at different locations and layers in healthy eyes of Saudi participants.

**Methods:**

Thirty-six randomly selected adults aged 27.0 ± 5.7 years who attended a Riyadh hospital from 2016 to 2017 were categorized into three groups: non-myopic (spherical equivalent refraction [SER], +1.00 to –0.50 diopters [D]), low myopic (SER, –0.75 to –3.00D), and moderate to high myopic (SER ≤ –3.25D). Full, inner, and outer retinal thicknesses were measured at nine locations by spectral-domain stratus optical coherence tomography (Optovue Inc., Fremont, CA, USA) and were compared according to refractive group and sex.

**Results:**

The mean SERs for the non-myopia, low myopia, and moderate to high myopia groups were 0.2 ± 0.6, –1.5 ± 0.5, and –7.5 ± 1.9 D, respectively. Refractive error, but not sex, had significant effects on the retinal layer thickness measurements at different locations (*P* < 0.05). The parafoveal and outer retinal layers were significantly thicker than the perifoveal and inner retina layers in all groups (*P* < 0.05). The full foveal thickness was higher and the full parafoveal and perifoveal regions were thinner in moderate to high myopic eyes than in the non-myopic eyes (*P* < 0.05), but were similar to those in the low myopic eyes (*P* > 0.05). The foveal thicknesses measured in the inner and outer layers of the retina were higher but the thicknesses measured at the inner and outer layers of the parafoveal and perifoveal regions were lower in moderate to high myopic eyes.

**Conclusion:**

There were regional differences in the retinal layer thicknesses of healthy Saudi eyes, which was dependent on the central refractions. This is important when interpreting retinal nerve fiber layer thicknesses in myopia and disease management in Saudi participants.

##  INTRODUCTION 

The global prevalence of myopia is increasing rapidly. Currently, 30% of the world population is myopic and almost 50% is projected to be so by 2050.^[1]^ In Asia, the prevalence of myopia is higher (approximately 80%) compared with that in other regions (approximately 25%).^[2,3,4]^ Uncorrected myopia is a major cause of visual impairment^[5]^ and the majority of people with visual impairment reside in developing countries, including Saudi Arabia.^[6]^

Individuals with myopia are at an increased risk of developing significant ocular comorbidities which can lead to retinal atrophy and possible blindness.^[7,8]^ Histological studies have shown significant reductions in scleral and retinal thicknesses as the degree of axial myopia increases,^[9,10]^ and these are responsible for the high incidence of retinal pathologies in high myopes.^[7,8][11]^ The enlarged globe and the elongated axial length of the myopic eyes lead to an ocular dimension that is stretched beyond its normal limit, which may lead to retinal thinning.^[12,13]^ Ocular conditions such as glaucoma, which are prevalent among individuals with highly myopic eyes, may also significantly affect the thickness of the inner retinal layers.^[12,13]^

Optical coherence tomography (OCT) offers a modern technique for in vivo measurements of retinal thickness and enables the assessment of the relationship between myopia and retinal thickness. This device can be used to correctly interpret and differentiate between an enlarged myopic optic disc^[14,15,16,17]^ and optic disc changes often seen in glaucomatous eyes. However, the refractive error of patients affects the measurement of retinal thickness and can be a confounding factor when diagnosing eye pathologies. Although studies have investigated the changes in retinal thickness in other populations,^[17,18,19,20,21,22,23,24,25,26,27,28]^ they have considered only the central retinal region or recruited individuals with high or pathologic myopia,^[17,27]^ and the results have been inconclusive^[17,18,19,20,21,22,23,24,25,26,27,28]^ due to the different methodologies used in the studies.^[17,18,19,20,21,22,23,24,25,26,27,28][29]^

Retinal thickness measurements by OCT vary according to the population being studied;^[30]^ thus, knowledge of such population-based differences in retinal thickness distribution is important in the evaluation, treatment, and follow-up of patients in a particular population and/or with various ocular pathologies.^[17,31]^ However, studies investigating the effects of refraction groups on retinal thickness measurements across different locations have shown different results.^[23,24,28]^

The purpose of the present prospective hospital study was to investigate the changes in retinal thickness measured at different retinal regions (foveal, perifoveal, and parafoveal) and layers (full, inner, and outer retina) in non-myopic and myopic participants. The second aim of this study was to examine the effects of refraction on the retinal thickness measurements across retinal regions and layers of the macula. Retinal thickness was assessed in myopic and non-myopic adults of similar ages by spectral-domain OCT, and the measurements were compared between groups. We hypothesized that there would be significant differences in retinal thicknesses measured at different locations by OCT between the myopic and non-myopic eyes of Saudi participants. Histopathology studies have shown that in myopia, the retina thins and degenerates, especially at the posterior pole;^[32]^ these changes in the retina of myopic eyes may reflect in retinal thickness measurements by OCT.

##  METHODS

### Study Population and Setting

From November 2016 to February 2017, 36 young adults (12 men [33.3%] and 24 women [66.7%]) were recruited at random for the present study. In order to eliminate possible selection bias if the clinicians were to choose the participants themselves, an optometry student who was not part of the investigation team approached participants from among those attending a public hospital facility in Saudi Arabia. The examiner taking all OCT measurements (FZ) was blinded to the refractive group of the participants. The participants were categorized into three groups: non-myopic (spherical equivalent refraction [SER] range of +1.00 to –0.50 diopters [D]), low myopic (SER, –0.75 to –3.00 D) and moderate to high myopic (SER ≤ –3.25D) groups. All participants had cylindrical refractions of < –1.00 D and underwent comprehensive eye examinations including distance visual acuity (VA) assessment, autorefraction, intraocular pressure (IOP) measurement using a non-contact tonometer, visual field testing, and dilated fundus examination. For all participants, only data from one eye, which was determined by random selection was used. The randomization process involved generating a series of random numbers from 1001 to 2001 through a Microsoft Excel spreadsheet. Each number was printed, folded, and inserted into a black box by a member of the clinical staff. A student clinician in charge of the data to be analyzed but blinded to the initial process of number generation selected a paper from the black box each time a participant's data was to be entered into the spreadsheet. A participant's right eye data were included in the analysis if the number picked was even (e.g., 1008) and the left eye data were included if the number was odd.

Ethics approval for this study was obtained from the College of Applied Medical Science, Research Ethics Committee for Human Participants, and the study was conducted in accordance with the Declaration of Helsinki as revised in the year 2000. Informed consent was obtained from all participants before their enrollment in this study [Table 1].

**Table 1:** Characteristics of study participants (n = 36 eyes)


	**Non-myopia**	**Low myopia**	**Moderate to high myopia**	***P*** **-values**
Sex, n (males/females)	12 (4/8)	12 (4/8)	12 (4/8)	0.32
Age, years	27.2 ± 5.3	27.3 ± 5.1	26.7 ± 6.9	0.59
SER, mean ± SD (range) (D)	0.18 ± 0.59 (+1.00 to –0.50)	–1.54 ± 0.54 (–0.75 to –3.00)	–7.50 ± 1.90 (–3.25 to –10.50)	< 0.001*
SER*, *spherical equivalent refraction; D, diopter; SD, standard deviation; n, number *Statistical significance from analysis of variance (ANOVA) Values are expressed as mean ( ± standard deviations)				

**Table 2:** Mean retinal thickness measurements (µm) and comparisons between groups at nine locations in non-, low, and moderate to high myopic healthy Saudi participants


**Retinal thickness locations**	**Non-myopia**	**Low myopia**	**Moderate to high myopia**	***P*** **-values**
Full retinal thickness at 1-mm zone	238.5 ± 8.4	253.4 ± 8.4	261.4 ± 8.1	< 0.0005 ${}^{a,b}$ , 0.071 ${}^{c}$
Full retinal thickness at parafovea	317.4 ± 14.2	295.8 ± 3.5	288.2 ± 5.5	< 0.0005 ${}^{a,b}$ , 0.143 ${}^{c}$
Full retinal thickness at perifovea	280.9 ± 11.3	274.0 ± 6.7	268.3 ± 5.3	0.138 ${}^{a}$ , 0.002 ${}^{b}$ , 0.296 ${}^{c}$
Inner retinal thickness at 1-mm zone	62.5 ± 3.8	65.9 ± 2.4	75.5 ± 4.0	0.065 ${}^{a}$ , < 0.0005 ${}^{b,c}$
Inner retinal thickness at parafovea	128.1 ± 2.8	119.4 ± 3.3	112.3 ± 5.2	< 0.0005 ${}^{a,b,c}$
Inner retinal thickness at perifovea	107.8 ± 2.9	96.9 ± 3.7	91.4 ± 5.3	< 0.0005 ${}^{a,b}$ , 0.007 ${}^{c}$
Outer retinal thickness at 1-mm zone	158.2 ± 3.9	164.6 ± 1.7	170.1 ± 4.0	< 0.0005 ${}^{a,b}$ , 0.001 ${}^{c}$
Outer retinal thickness at parafovea	180.8 ± 3.1	177.4 ± 4.1	171.3 ± 2.5	0.048 ${}^{a}$ , < 0.0005 ${}^{b,c}$
Outer retinal thickness at perifovea	175.3 ± 4.3	171.5 ± 2.6	167.3 ± 2.8	0.025 ${}^{a}$ , < 0.0005 ${}^{b}$ , 0.014 ${}^{c}$
SD, standard deviation ${}^{a}$ The mean difference is significant at the 0.05 level for non-myopia versus low myopia ${}^{b}$ The mean difference is significant at the 0.05 level for non-myopia versus moderate-to-high myopia ${}^{c}$ The mean difference is significant at the 0.05 level for low myopia versus moderate-to-high myopia. Results are post hoc analysis with Bonferroni correction *P*-values are *post hoc* results of analysis of variance (ANOVA)				

### Inclusion and Exclusion Criteria

Participants were included in this study if the best-corrected VA was better than 6/9 in each eye and the difference in SER between eyes was < 1.00 D. The exclusion criteria were history of amblyopia, any ocular disease, surgery and/or medications, ocular trauma, in-cyclotorsion or ex-cyclotorsion of the eye, anisometropia (difference in SER between eyes ≥ 1.00 D), systemic diseases with ocular implications, the best-corrected VA worse than 6/9, IOP > 21 mmHg, a personal or family history of glaucoma or any neurologic condition with visual field effects or any other optic neuropathy.

### OCT Protocol

**Figure 1:**
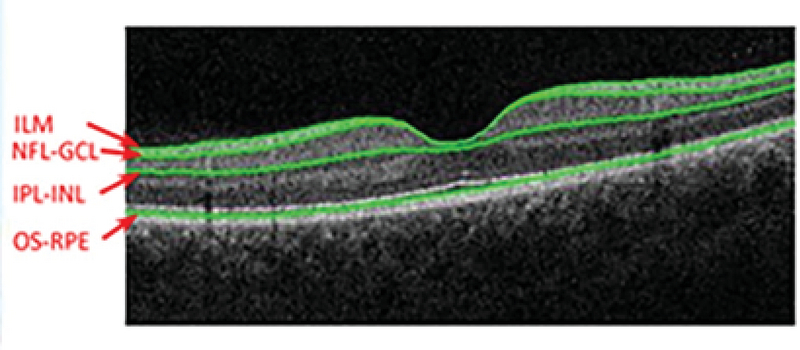
Full retinal layer scanning of a participant (right). RPE, retinal pigment epithelium; IPL, inner plexiform layer; ILM, inner limiting membrane; GCL, ganglion cell layer.

**Figure 2:**
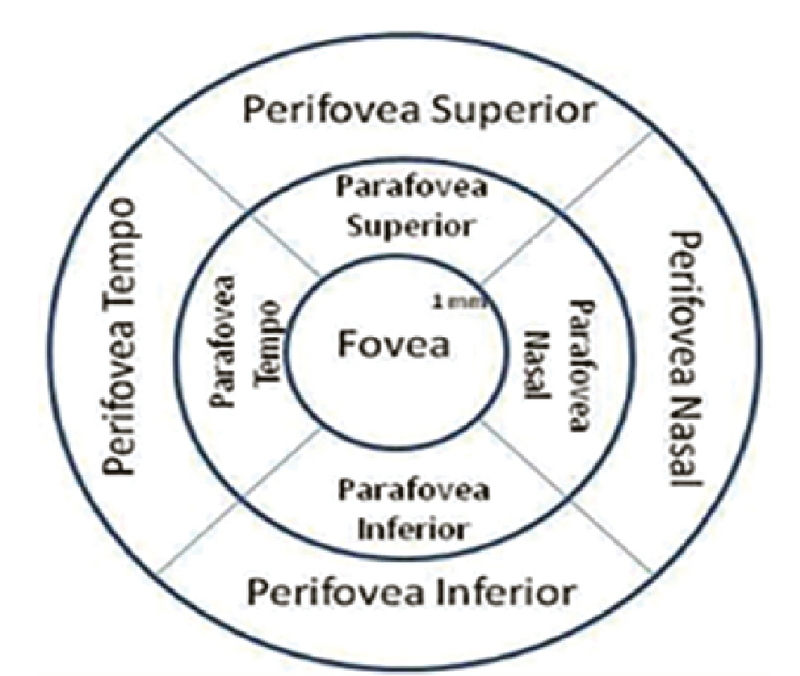
The nine zones of the Optovue RTVue spectral-domain ocular coherence tomography (SD-OCT) retinal map defined by standard the Early Treatment Diabetic Retinopathy Study (ETDRS) grid.

Retinal thickness was measured using the RTVue Spectral Domain OCT (Optovue Inc., Fremont, CA, USA). The instrument obtains retinal thickness information using the MM6 scan protocol [Figure 1]. For all participants, both eyes were dilated by administering 1% tropicamide and 2.5% phenylephrine hydrochloride three times over a 10-min period before the OCT procedure. Only OCT scans of sufficient quality (signal ≥ 50% of maximum strength, absence of imaging artifacts or distortions) were used. If the quality of the OCT scans was insufficient, replicate measurements were taken. The instrument uses 12 radial lines, each 6 mm long, that are centered at the fovea to provide 1,024 A-scans, each of the full retinal thickness. Using the RTVue-100 instrument software (version 4.0.5.39; Optovue, Inc., Fremont, CA), the full retinal thickness was measured automatically at three concentric zones: a circle of 1 mm diameter centered at the fovea, a parafoveal region with an inner and an outer diameter of 1 mm and 3 mm, respectively, and a perifoveal region with an inner and an outer diameter of 3 mm and 6 mm, respectively. The nine zones of the retinal thickness shown in Figure 2 were compared between the three refractive groups. The boundaries for segmentation by the software included: total retinal (TR) thickness, from the inner limiting membrane to the outer RPE; inner retinal thickness, from the inner limiting membrane to the outer boundary of inner plexiform layer; outer retinal thickness, from the outer boundary of inner plexiform layer to the outer RPE.

### Statistical Analysis

Data were analyzed using the IBM SPSS Statistics for Windows, version 22.0 (IBM Corp., Armonk, NY, USA). *P* < 0.05 was considered significant. All data were analyzed for normality using the Shapiro–Wilk test and the results were presented using descriptive statistics. Pearson correlation coefficients were used to determine the relationship between retinal thicknesses at different locations. Independent *t*-test analysis, Fisher’s exact test and Chi-square analysis, and analysis of variance (ANOVA) with “location” as a between-subject factor and “sex” as a within-subject factor were performed where appropriate. Where significant differences were found,* post hoc* analysis was conducted after applying Bonferroni correction for multiple comparisons.

##  RESULTS

**Figure 3:**
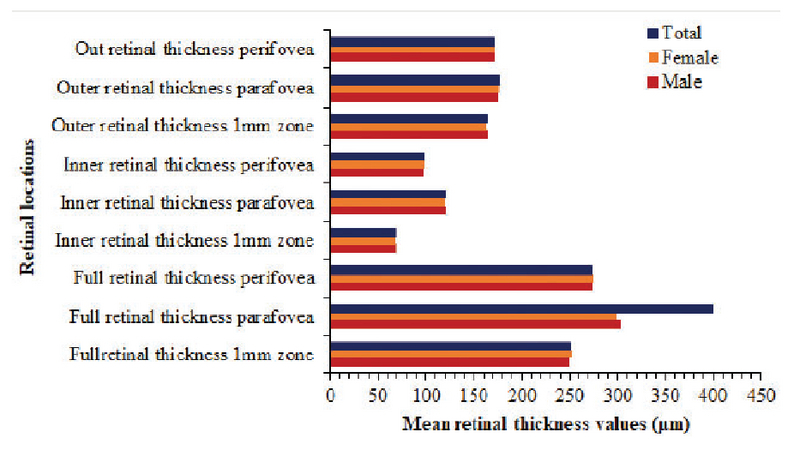
Mean retinal thickness measurements (µm) at different locations in male and female Saudi participants.

A summary of the demographic data for the non-, low, and high myopic participants and the results of the comparative analysis are provided using descriptive statistics in Table 1. The participants were matched for age (*P* = 0.50, unpaired *t*-test) and sex (*P* > 0.05, Fisher's test) but each group included more female than male participants (57% versus 43%). The mean SER for all participants ranged from +1.00 D to –10.50 D, and male participants were more myopic than female participants (mean ± SD, –6.18 ± 2.24 D versus –4.01 ± 1.75 D, *P* < 0.001) in this study. Figure 3 shows the mean thicknesses across retinal locations for all participants. Sex was not significantly correlated with the retinal thickness at each of the locations and did not affect the measured thicknesses at any of the nine locations (*P* > 0.05, for all results of independent *t*-test analysis).

Table 2 shows the full and regional retinal thickness values for each group and the differences between groups. Significant differences were observed in the retinal thicknesses measured across the nine locations in each group (repeated measures ANOVA, *P* < 0.001). The full retinal thickness at the parafoveal region was the highest among the refractive groups with averages of 317.4 µm, 295.8 µm, and 288.2 µm in the non-, low, and moderate to high myopic groups, respectively. In contrast, the inner retinal 1 mm zone had the lowest thickness, with an average of 62.5 µm, 65.9 µm, and 75.5 µm, in the non-, low, and moderate to high myopic groups, respectively.

**Figure 4:**
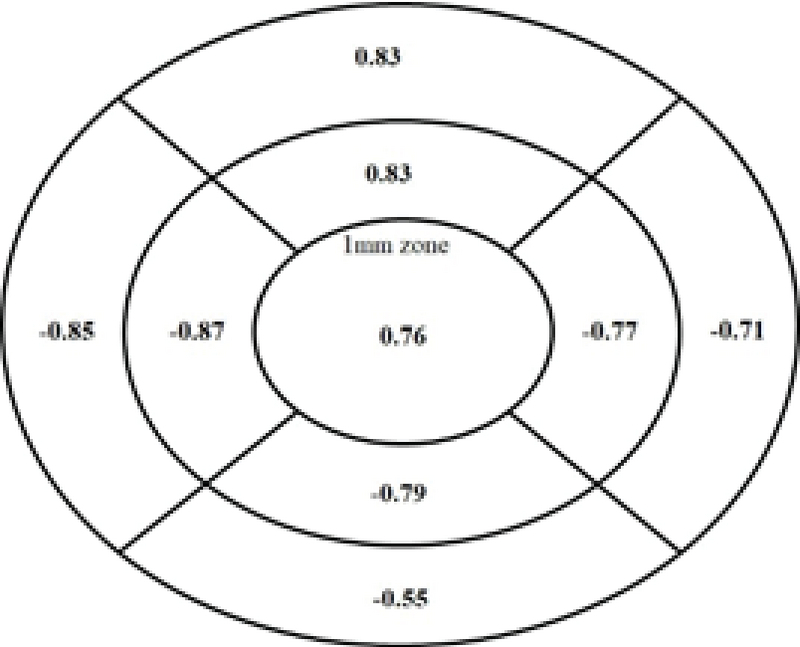
Pearson correlation coefficients (r) between refractive errors and retinal thicknesses (µm) at nine retinal locations using the Early Treatment Diabetic Retinopathy Study (ETDRS) grid.

Figure 4 shows the correlation coefficients (r) between retinal thickness and refractive error groups for the nine retinal locations representing each segment of the nine field Early Treatment Diabetic Retinopathy Study (ETDRS) grid. All correlations between refractive error group and retinal thicknesses at all locations were significant (*P* < 0.05). The refractive error group was strongly correlated with retinal thicknesses at all locations (r ≥ 0.70) except for the full retinal thickness at the perifoveal region, which showed moderate correlation (r = –0.55).

Significant differences were observed in mean retinal thicknesses between the refractive groups (*P *< 0.0001 for each location), and post hoc analysis showed no differences in the full retinal thickness measured at the 1 mm zone, the para-fovea and peri-fovea regions, and between the low and the moderate to high myopic groups (*P *= 0.07, 0.14, 0.30, respectively), but there were significant differences in the full retinal thickness at the 1 mm and at parafovea zones (both, *P* < 0.001) but not in the perifovea zone (*P *= 0.14) when non-myopic eyes were compared to low myopic eyes. We observed significant differences in mean retinal thickness values between the non- and moderate to high myopic groups (*P* < 0.05). At the 1 mm zone, the retina was significantly thicker but was significantly thinner at every other location in the moderate to high myopic group than that in the non-myopic group [Table 2]. Overall, the mean retinal thickness was significantly lower in the para- and perifoveal regions and higher in the 1 mm zone in the moderate to high myopic group than in the other groups [Table 2].

##  DISCUSSION 

This study measured retinal thickness in young, non-myopic Saudi participants by spectral-domain OCT and compared the measurements with those obtained in age-matched groups of participants with low and moderate to high myopia. We found that across the refractive groups, the parafoveal region was significantly thicker than the perifoveal retina and both were thicker than the fovea (1 mm zone). The full foveal thickness was significantly higher and the full parafoveal and perifoveal retinal thicknesses were significantly lower in the moderate to high myopic group than in the non-myopic and low-myopic groups. We observed a significant effect of refractive error on the measurements of retinal thickness across different regions and locations. The full retinal thickness measured at the perifoveal, parafoveal, and 1 mm central zone differed significantly between the moderate to high myopic group and the non-myopic group across all locations. The thicknesses measured at the perifoveal and parafoveal regions (including the inner and outer layers of the retina), and those measured at the 1 mm zone, were significantly higher and lower, respectively, in the non-myopic group than in the moderate to high myopic group [Table 3].

The present study found that the degree of myopia was significantly related to the retinal thickness in the study cohort. The retinal thicknesses at the parafoveal and perifoveal regions rapidly decreased as the degree of myopia increased. Similar findings have previously been reported.^[18][23,24,28][33]^ The retinal thinning observed in myopic eyes has been linked to mechanical stretching of the sclera due to the increased axial length of the enlarged myopic eyeball. The perifoveal region is also less resistant to traction and stretching forces and is devoid of large blood vessels,^[34]^ which could partly explain these findings. A Chinese study that recruited individuals with different refractions reported significant changes in macular thickness that varied with the degree of myopia. However, the patterns of change varied according to the different macular areas and layers.^[28]^ In line with our findings, the authors also reported significant positive (slightly weaker than our study finding) correlations between SER and full parafoveal (r = 0.4, *P* < 0.001) and perifoveal thicknesses (r = 0.5, *P* < 0.001).^[28]^

The fovea was thicker in the moderate to high myopic group than in the other refractive groups, similar to the findings of previous studies.^[18,23,24]^ This observation has been attributed in part to genetic factors, particularly in myopic eyes, which cause increased thickness in the inner nuclear layer (INL), inner plexiform layer (IPL), and ganglion cell layer.^[35]^ Lam et al,^[23]^ reported preservation of the retinal thickness in the most central foveal region in highly myopic eyes compared to low myopic eyes and attributed this difference to tangential traction resulting from the internal limiting membrane or posterior vitreous cortex.^[22,36]^ Similar to another study,^[36]^ we found that the perifoveal retinal region was thinner than the parafoveal retinal region across the refractive groups.

The male participants in this study were generally more myopic than the female participants but sex did not significantly affect the refraction differences between the groups or the measured retinal thicknesses across the nine retinal locations (*P* > 0.05). Contrary to our results, studies from China^[37,38]^ found significant differences between the sexes in absolute central retinal thickness, noting lower minimum foveal, average foveal (1 mm), and average inner ring macular thicknesses in women than those in men.^[23]^ Although these studies had larger sample sizes compared to our study, the population also differed, with other studies including mostly Chinese participants. Other studies, which recruited Europeans in Netherlands^[39]^ and a mixture of Europeans, Africans, and African Americans living in the US^[40]^ also reported no effect of sex on retinal thickness as measured by OCT,^[39,40]^ suggesting that the differences between the studies on the effects of sex may be due to the differences in the study populations.

The limitations of this study include the small sample size. This was largely due to the strict selection criteria and the method of participant selection. Due to the unavailability of ocular biometry measurements, we were unable to assess the effects of axial length on retinal thickness. Therefore, it remains unclear whether the differences were due to axial or refractive myopia. However, the relationship between axial length and retinal thickness at various locations remains controversial: Lam et al^[23]^ reported a negative correlation between total macular thickness and axial length and a positive correlation between foveal thickness and axial length, while Huynh et al^[41]^ showed retinal thinning with increasing axial length in the outer and inner macular regions but not in the central macula.

This study provided what we believe to be the first evidence of regional variations in retinal thicknesses within the Saudi population and contributes to the growing body of evidence on the diagnostic use of OCT in myopia. However, the study was limited by the narrow range of subjects' ages and the exclusion of participants with diseases such as ocular hypertension, glaucoma, and diabetes, where precise measurement of central corneal thickness is also important. Further studies including Saudi participants with a wider age range and those diagnosed with ocular diseases are needed to confirm the findings of the present study.

In conclusion, we observed regional variations in retinal thickness measurements, with greater thickness at the parafoveal region than in the perifoveal and central regions (1 mm) across refractive groups. The differences in retinal thickness measurements among the groups across locations were not dependent on the participant's sex. Non-myopic eyes had significantly thicker retinas at the peri- and para-foveal regions but thinner retinas at the 1 mm foveal zone compared with the myopic eyes. In contrast, the fovea was significantly thicker at the 1 mm zone and thinner at other locations around the fovea of moderate to high myopes compared with non-myopes. Evaluation of retinal thickness in disease conditions such as glaucoma and macular degeneration should be interpreted in the light of the degree of refractive error and the region of measurement.

##  Financial Support and Sponsorship

Nil.

##  Conflicts of Interest

There are no conflicts of interest.
